# A population-based survey of self-reported and IIEF-defined erectile dysfunction among adult men in the United States in 2021

**DOI:** 10.1186/s12889-025-24808-4

**Published:** 2025-11-27

**Authors:** Caroline Amand, Sabine Tong, Anne-Laure Tardy, Thomas McGraw, Andrew Stewart, Mario Cruz-Rivera

**Affiliations:** 1 Sanofi, Neuilly-sur-Seine, France; 2 Sanofi, Bridgewater, NJ USA; 3 Sanofi, Cambridge, MA USA

**Keywords:** Erectile dysfunction, IIEF-5, Survey, Prevalence

## Abstract

**Background:**

Erectile dysfunction (ED) is the most common male sexual medical condition worldwide, but assessing its prevalence accurately is made difficult by the diversity of methodologies utilised. This study used the abridged 5-item International Index of Erectile Function (IIEF-5) questionnaire, together with estimates based on self-reported ED and ED treatments, to assess ED prevalence in the US.

**Methods:**

Analyses were undertaken by age groups: 18–34 years; 35–49 years; 50–64 years; ≥65 years, with further assessments by socioeconomic status (SES), lifestyle and health conditions associated with ED. Continuous variables, categorical variables and multivariate logistic regression models were used.

**Results:**

Sexually active men aged ≥18 years, from all 50 USA states, Washington, DC, and Puerto Rico, were recruited through the Evidation Health, Inc. (San Mateo, CA, USA; evidation.com) health-tracking platform (n=3,289). ED prevalence was found to increase with age, from 26.0% (18–34 years) to 62.5% (≥65 years) based on the IIEF-5 questionnaire, from 19.3% to 60.0% based on ED self-perception, and from 2.6% to 29.2% based on current treatments. Other factors that were found to increase the risk of ED included low SES; health conditions, such as diabetes, chronic pain, mental health conditions; and being of Black or Asian heritage.

**Conclusion:**

This study corroborates established knowledge that ED prevalence increases with age, is strongly associated with comorbidities, and is influenced by socioeconomic factors. Importantly, our findings reveal a notable discrepancy between IIEF-5-defined ED and self-reported symptoms, highlighting a critical public health consideration: ED often serves as an early warning sign for more serious underlying health conditions. This underscores the importance of improved screening and awareness in clinical practice.

**Supplementary Information:**

The online version contains supplementary material available at 10.1186/s12889-025-24808-4.

## Introduction

Erectile dysfunction (ED) includes an impairment in the arousal phase of sexual response and is defined as the consistent or recurrent inability to attain and/or maintain a penile erection sufficient for sexual satisfaction, including satisfactory sexual performance [1, 2]. There are many factors associated with an increased risk of ED, which can be socio-demographic, such as older age [3], a low socio-economic status (SES) [4] and a low level of education [5], or lifestyle choices such as tobacco, alcohol, recreational drug use and lack of physical exercise [4–6]. Comorbidities are also associated with ED, for example, obesity, diabetes, cardiovascular diseases, high blood pressure, dyslipidaemia and low testosterone [4, 7–10]. Additionally, multiple social and psychological factors are associated with ED, such as sexual performance anxiety, relationship problems, depression, anxiety and stress [3, 10–12]. Together, these factors can cause broad socio-economic consequences in people with ED, compared to the population without ED, such as significantly higher work absenteeism, lower overall work productivity and impairment of activities [13].

ED is the most common sexual medical condition in males worldwide [3] and in 2012, affected about 30 million men in the USA [14]. Worldwide, in 1995, the prevalence rate of ED was estimated to be over 152 million and was projected to reach 322 million in 2025, calculated using the prevalence rate of two large studies, the Massachusetts Male Aging Study (MMAS) [15] and the United Nations-projected male population distributions [16]. It is worth noting however, that in some cases prevalence rates need to be interpreted with caution. A review of epidemiology studies in ED showed that prevalence rates can be affected by many factors including age [8], study design (local, national, international or multiple site studies) study type (retrospective or prospective) and data type (claims data and/or electronic health records)) [4, 8, 10, 13, 17, 18]. Another influence on prevalence rate includes the method used to identify ED [19]. Methods available for measuring ED range from simple unvalidated questions, for example relating to satisfactory sexual intercourse [5, 10, 20, 21], to specific surveys such as the MMAS [15], or validated self-administered questionnaires, such as the abridged 5-item International Index of Erectile Function (IIEF-5) questionnaire) [22, 23].

Undertreatment of ED as well as a lack of clinical data is an issue; stigma, embarrassment and a lack of understanding of possible treatments for ED mean that men often refrain from discussing ED issues with their healthcare provider [11, 12, 24]. As well, ED prevalence may be higher in some ethnic groups than others, and differing cultural values can affect the likelihood of men seeking treatment [5]. It has been shown that anonymous surveys of patients, such as the IIEF-5 questionnaire, may produce a more precise measure of ED prevalence than direct questions from healthcare professionals about self-perceived ED [25, 26].

The main objective of this study was to estimate the prevalence of ED by age group, based on signs of ED using the IIEF-5 questionnaire score (referred to from hereon in as IIEF-5-defined ED). The second objective was to estimate the prevalence of ED using questions based on self-perception and current ED treatment use. Other objectives were to evaluate the demographic and clinical characteristics of participants with and without IIEF-5-defined ED, to determine whether sociodemographic and clinical characteristics can predict IEEF-5-defined ED, and to assess the distribution of ED severity and the distribution of self-perception and management of participants with IIEF-5-defined ED.

## Methods

### Study design and data source

This was a cross-sectional study using data from a Men’s Sexual Health Survey administered to study participants throughout December 2021. Participants were recruited through Evidation Health, Inc., a real-world, health-tracking platform covering 50 states in the USA, Washington DC, and Puerto Rico that generates Person-generated health data (PGHD) [27].

The Men’s Sexual Health Survey is a one-time, population-based questionnaire specifically developed by Sanofi (Bridgewater, USA) and Evidation Health, Inc. (San Mateo, CA, USA; evidation.com). It consists of three sections capturing sociodemographic characteristics, lifestyle and behaviours, and health conditions, as well as ED-specific questions, including ED self-perception, current ED treatment use, and the IIEF-5 questionnaire to evaluate ED diagnosis and severity [28]. To mitigate the effects of selection bias, two sampling methodologies were used to recruit participants from the Evidation panel: general convenience sampling and re-sampling based on a proportionally weighted distribution of race derived from the 2021 US Census population estimates [29].

### Participants

The study population included men aged over 18 years, living in the USA and Puerto Rico, and able to speak, read and understand English. All the study participants provided written informed consent prior to survey completion. Participants who had not been sexually active for the last 6 months (based on an IIEF-5 questionnaire score of < 5with SUM[Q2 to Q5] = 0) were excluded from the analyses.

### Variables

All variables included in the analyses were collected through the Men’s Sexual Health Survey and gathered at the time of the questionnaire. Sociodemographic data collected included sex, age, geographical region, race/ethnicity, education level, employment status, annual household income and relationship status. For lifestyle behaviours, data related to alcohol consumption, smoking and exercising frequency were collected. Medical conditions that occurred from a predefined list were considered.

Data related to erectile dysfunction diagnosis were evaluated based on answers to the IIEF-5 section of the Men’s Sexual Health Survey taking into consideration sexual activity over the past 6 months [22, 28]. A total score < 5 with SUM(Q2 to Q5) = 0 defined “not sexually active”, a total score ranging from 2 to 21 with SUM(Q2 to Q5) > 0 defined a “diagnosis of ED” and a total score > 21 defined an “absence of ED”. Data related to ED severity were collected among sexually active males (IIEF total score > 5) and were defined, according to IIEF-5 score as follows: “severe ED” (total IIEF-5 score 2–7); “moderate ED” (total IIEF-5 score 8–11); “mild to moderate ED” (total IIEF-5 score 12–16) and “mild ED” (total score 17–21). Data related to perception and management of ED (“I have not experienced ED”, “I have experienced ED, but have not spoken to a healthcare provider or sought a diagnosis”, “I have spoken to a healthcare provider about ED, but have not received a diagnosis” and “I have been diagnosed by a healthcare provider as having ED, but do not have a prescription”) were self-reported by the patients via the survey. Data related to the current use of oral prescription medications to treat ED (Viagra^®^ [Pfizer], Levitra^®^ [Bayer], Staxyn^®^ [Bayer], Cialis^®^ [Lilly] and Stendra^®^ [Mitsubishi Tanabe Pharma]) were self-reported by the patients.

### Statistics

All the analyses were stratified by age groups aligned with previous studies [30]: young adults (18–34 years); adults (35–49 years); older adults (50–64 years); elderly (≥ 65 years). Continuous variables were summarised using means, standard deviations (SDs), medians and interquartile range (Q1–Q3). Categorical variables were summarised using counts and percentages. ED prevalence was calculated for each age group by dividing the number of participants with ED by the total number of participants, and 95% confidence intervals (CIs) were tabulated for weighted prevalence estimate (using the racial/ethnic distribution from the 2021 USA Census by age group). Prevalence estimates based on the IIEF-5 questionnaire, self-perception questions and current ED treatment questions were assessed by contrasting the distribution across age groups. Among those with self-defined ED, distribution by IIEF-defined ED severity, self-perception and management of ED, and current ED medication use, was described. Multivariate logistic regression models were performed for each age group to evaluate predictive signs of self-defined ED through the IIEF-5 questionnaire versus those without ED. Model covariates included all sociodemographic, clinical and lifestyle characteristics. Stepwise selection was used, and odds ratios (ORs), 95% CIs and p-values were calculated. To assess the impact of possible selection bias on the study findings, a sensitivity analysis was performed that examined ED prevalence (based on IIEF-5 questionnaire scores) across SES levels (low, middle and high). A weighted SES score was calculated for each participant and then categorised by age group based on the statistical distribution: low (lower 25% of the distribution of the SES index), middle (25–75% of the distribution) and high (upper 75% of the distribution) [31].

## Results

### Study population (cohort of all sexually active men)

**Fig. 1:**
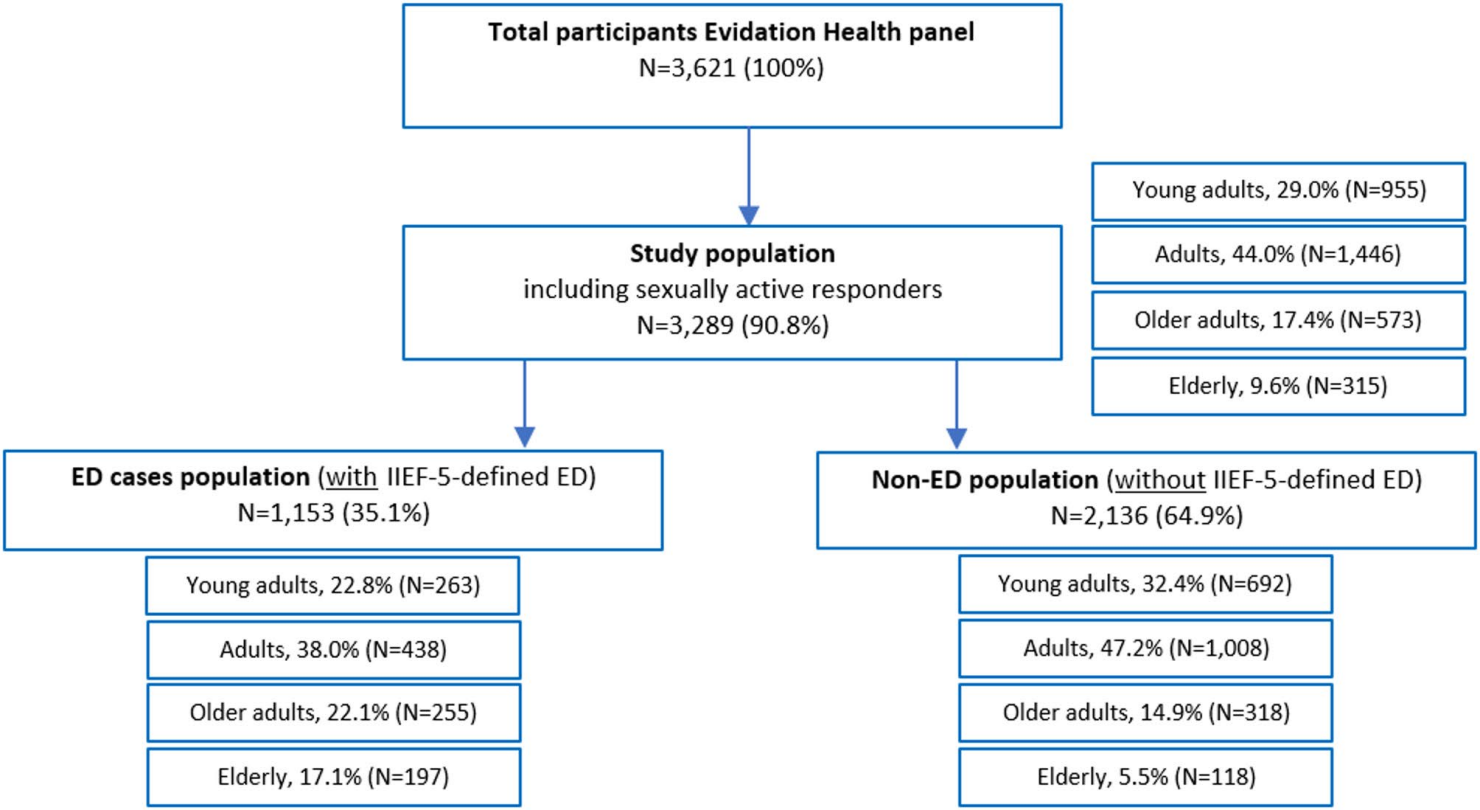
Participant distribution. ED, erectile dysfunction; IIEF-5, five-item International Index of Erectile Function

In total, 3,621 participants were recruited through the Evidation Health, Inc. platform. Of these, 280 (7.7%) were excluded due to being identified as having been sexually inactive within the last 6 months, and 52 (1.4%) were excluded owing to inconsistent responses. Of the remaining 3,289 (90.8%) sexually active responders, 29.0% (*N* = 955) were young adults, 44.0% (*N* = 1,446) adults, 17.4% (*N* = 573) older adults, and 9.6% (*N* = 315) were elderly. This cohort was used to evaluate ED prevalence (Fig. 1**).**

Selected sociodemographic and lifestyle characteristics, and health condition outcomes of the weighted characteristics, are shown in Tables 1 and 2 and Supplementary Table 1.

**Table 1:** Sociodemographic characteristics of the sexually active men population by age group (%weighted)

	Young adult 18 to 34 years (*N* = 955)	Adults 35 to 49 years (*N* = 1,446)	Older adults 50 to 64 years (*N* = 573)	Elderly ≥ 65 years (*N* = 315)	Overall (*N* = 3,289)
**Age (years)**,** mean (SD)**	30 (3.5)	41 (4.2)	55 (4.1)	69 (4.1)	43 (12.9)
**Region**,** n (%)**					
Northeast	190 (20.3%)	304 (21.2%)	92 (16.2%)	59 (18.7%)	645 (19.8%)
South	355 (37.1%)	534 (37.0%)	240 (42.0%)	124 (39.4%)	1,253 (38.1%)
Midwest	222 (24.1%)	352 (24.7%)	146 (25.2%)	80 (25.4%)	800 (24.7%)
West	180 (17.7%)	255 (17.2%)	94 (16.4%)	52 (16.5%)	581 (17.1%)
Missing	8 (0.9%)	1 (0.1%)	1 (0.2%)	0 (0.0%)	10 (0.3%)
**Race**,** n (%)**					
White	607 (63.6%)	1,023 (70.7%)	438 (76.4%)	267 (84.8%)	2,335 (71.0%)
Black	117 (12.3%)	171 (11.8%)	64 (11.2%)	25 (7.9%)	377 (11.5%)
Asian	78 (8.2%)	103 (7.1%)	29 (5.1%)	14 (4.4%)	224 (6.8%)
Other/mixed Race	132 (13.8%)	136 (9.4%)	37 (6.5%)	9 (2.9%)	314 (9.5%)
Missing	21 (2.2%)	13 (0.9%)	5 (0.9%)	0 (0.0%)	39 (1.2%)
**Education level**,** n (%)**					
Grade school	1 (0.1%)	1 (0.1%)	0 (0.0%)	0 (0.0%)	2 (0.1%)
Some high school	5 (0.5%)	9 (0.6%)	6 (1.0%)	3 (1.0%)	23 (0.7%)
Completed high school	39 (4.1%)	57 (3.9%)	43 (7.5%)	20 (6.3%)	159 (4.8%)
Some college	131 (13.7%)	221 (15.3%)	98 (17.1%)	47 (14.9%)	497 (15.1%)
Trade/technical school	28 (2.9%)	52 (3.6%)	31 (5.4%)	14 (4.4%)	125 (3.8%)
Completed college	492 (51.5%)	681 (47.1%)	248 (43.3%)	145 (46.0%)	1,566 (47.6%)
Graduate studies/ advanced degree	256 (26.8%)	417 (28.8%)	147 (25.7%)	83 (26.3%)	903 (27.5%)
Other	0 (0.0%)	2 (0.1%)	0 (0.0%)	0 (0.0%)	2 (0.1%)
I prefer not to answer	3 (0.3%)	6 (0.4%)	0 (0.0%)	3 (1.0%)	12 (0.4%)
**Current employment status**,** n (%)**					
Employed full-time	813 (85.1%)	1,334 (92.3%)	462 (80.6%)	65 (20.6%)	2,674 (81.3%)
Employed part-time	66 (6.9%)	39 (2.7%)	22 (3.8%)	27 (8.6%)	154 (4.7%)
Not employed and not looking for employment	24 (2.5%)	19 (1.3%)	6 (1.0%)	2 (0.6%)	51 (1.6%)
Not employed but looking for employment	28 (2.9%)	20 (1.4%)	10 (1.7%)	1 (0.3%)	59 (1.8%)
Retired	2 (0.2%)	15 (1.0%)	61 (10.6%)	218 (69.2%)	296 (9.0%)
Other	22 (2.3%)	19 (1.3%)	12 (2.1%)	2 (0.6%)	55 (1.7%)
**Annual household income (USD)**,** n (%)**					
<$25k	70 (7.3%)	31 (2.1%)	10 (1.7%)	11 (3.5%)	122 (3.7%)
$25k–$34,999	62 (6.5%)	62 (4.3%)	18 (3.1%)	12 (3.8%)	154 (4.7%)
$35k–$49,999	110 (11.5%)	100 (6.9%)	36 (6.3%)	27 (8.6%)	273 (8.3%)
$50k–$74,999	212 (22.2%)	243 (16.8%)	94 (16.4%)	60 (19.0%)	609 (18.5%)
$75k–$99,999	165 (17.3%)	266 (18.4%)	111 (19.4%)	63 (20.0%)	605 (18.4%)
$100k–$149,999	191 (20.0%)	385 (26.6%)	133 (23.2%)	71 (22.5%)	780 (23.7%)
≥$150k	102 (10.7%)	281 (19.4%)	128 (22.3%)	43 (13.7%)	554 (16.8%)
I prefer not to answer	43 (4.5%)	78 (5.4%)	43 (7.5%)	28 (8.9%)	192 (5.8%)
**Relationship status**,** n (%)**					
Married	474 (49.6%)	1,077 (74.5%)	442 (77.1%)	255 (81.0%)	2,248 (68.3%)
Single, not dating	148 (15.5%)	107 (7.4%)	52 (9.1%)	23 (7.3%)	330 (10.0%)
Single, in a steady relationship, but not living with a partner	93 (9.7%)	60 (4.1%)	17 (3.0%)	10 (3.2%)	180 (5.5%)
Single, dating, but not living with a partner	90 (9.4%)	85 (5.9%)	26 (4.5%)	8 (2.5%)	209 (6.4%)
Living with a partner, but not married	148 (15.5%)	111 (7.7%)	33 (5.8%)	16 (5.1%)	308 (9.4%)

Responses were weighted using the racial/ethnic distribution from the 2021 USA Census by age group

**Table 2:** Lifestyle and behaviour characteristics of the sexually active men population by age group (% weighted)

	Young adult 18 to 34 years (*N* = 955)	Adults 35 to 49 years (*N* = 1,446)	Older adults 50 to 64 years (*N* = 573)	Elderly ≥ 65 years (*N* = 315)	Overall (*N* = 3,289)
Alcohol frequency, n (%)					
Never	161 (16.9%)	267 (18.5%)	126 (22.0%)	71 (22.5%)	625 (19.0%)
Once a month or less frequently	238 (24.9%)	371 (25.7%)	146 (25.5%)	77 (24.4%)	832 (25.3%)
2–4 times a month	280 (29.3%)	368 (25.4%)	126 (22.0%)	72 (22.9%)	846 (25.7%)
2–3 times a week	200 (20.9%)	281 (19.4%)	100 (17.5%)	44 (14.0%)	625 (19.0%)
≥ 4 times a week	76 (8.0%)	159 (11.0%)	75 (13.1%)	51 (16.2%)	361 (11.0%)
Smoking status, n (%)					
I’ve never smoked cigarettes	774 (81.0%)	1,028 (71.1%)	424 (74.0%)	191 (60.6%)	2,417 (73.5%)
I used to smoke cigarettes, but have quit smoking	127 (13.3%)	323 (22.3%)	120 (20.9%)	115 (36.5%)	685 (20.8%)
I smoke cigarettes a few days per month	21 (2.2%)	29 (2.0%)	4 (0.7%)	0 (0.0%)	54 (1.6%)
I smoke cigarettes a few days per week	8 (0.8%)	17 (1.2%)	8 (1.4%)	1 (0.3%)	34 (1.0%)
I smoke cigarettes every day	25 (2.6%)	49 (3.4%)	17 (3.0%)	8 (2.5%)	99 (3.0%)
Exercise frequency, n (%)					
Never	17 (1.8%)	53 (3.7%)	19 (3.3%)	10 (3.2%)	99 (3.0%)
Once a month or less frequently	114 (11.9%)	151 (10.4%)	56 (9.8%)	17 (5.4%)	338 (10.3%)
2–4 times a month	183 (19.2%)	262 (18.1%)	76 (13.3%)	30 (9.5%)	551 (16.8%)
2–3 times a week	289 (30.3%)	394 (27.2%)	155 (27.1%)	70 (22.2%)	908 (27.6%)
≥ 4 times a week	352 (36.9%)	586 (40.5%)	267 (46.6%)	188 (59.7%)	1,393 (42.4%)

Responses were weighted using the racial/ethnic distribution from the 2021 USA Census by age group

Most participants were White (71.0%), had completed a college-level education (47.6%), and most of those < 65 years old were employed full time (81.3%). The highest proportion of participants had an annual household income of $100,000–149,999 per year, with a higher proportion of young adult participants earning more than $75,000 in annual household income compared to the other groups. The proportion of participants who were married increased with each age group. A slightly higher proportion of older adults (22.0%) and the elderly participants (22.5%) reported never drinking alcohol compared to younger adults (16.9%) and adults (18.6%). Drinking alcohol 4 or more times per week increased slightly with advancing age, then decreased among participants aged ≥ 65 years. Most participants in every age group were never smokers (young adults: 81.6%; adults: 71.2%; older adults: 73.8%; elderly: 60.6%). The proportion of participants who reported having at least one of the selected health conditions of interest increased with advancing age, ranging from 26.3% of young adults who reported having at least one health condition compared to 41.2% of adults, 65.2% of older adults and 76.8% of elderly participants. The proportion of respondents reporting hypertension, high cholesterol and diabetes also increased by age group. Conversely, mental health conditions were more prevalent among younger participants compared to those aged > 50 years of age.

## Prevalence of ED

Prevalence of ED was defined using the cohort of all sexually active men according to IIEF-5 score, patient self-perception and current use of ED treatment. ED prevalence is provided per the four age groups and is presented in Table 3. Overall, ED prevalence increased across age groups independently of the assessment method. Prevalence estimates, based on the IIEF-5, were the highest (young adults: 26.0%; adults: 30.3%; older adults: 44.6%; elderly: 62.5%), followed by prevalence estimates based on patient self-perception and management (young adults: 19.3%; adults: 27.0%; older adults: 39.2%; elderly: 60.0%). Use of current ED treatment had the lowest prevalence estimates in every age group (young adults: 3.3%; adults: 6.8%; older adults: 16.7%; elderly: 29.2%).

**Table 3:** Prevalence (weighted) of ED based on the IIEF-5 questionnaire, self-perception, and current treatment by age group in sexually active men

		Young adults 18 to 34 years (*N* = 955)	Adults 35 to 49 years (*N* = 1,446)	Older adults 50 to 64 years (*N* = 573)	Elderly > 65 years (*N* = 315)
**Based on IIEF-5 questionnaire**	n (%)	263 (26.0%)	438 (30.3%)	255 (44.6%)	197 (62.5%)
	95% CI	23.3–29.0	28.0–32.8	40.5–48.7	57.0–67.7
**Based on self-reporting**	n (%)	188 (19.3%)	392 (27.0%)	225 (39.2%)	189 (60.0%)
	95% CI	16.9–22.0	24.7–29.4	35.2–43.3	54.5–65.3
**Based on current ED medication**	n (%)	35 (3.3%)	98 (6.8%)	95 (16.7%)	92 (29.2%)
	95% CI	2.4–4.7	5.6–8.2	13.8–20.0	24.4–34.5

Responses were weighted using the racial/ethnic distribution from the 2021 USA Census by age group

The numbers of participants in each ED group (IIEF-5 questionnaire, self-perception, ED treatment) are not mutually exclusive

*ED* erectile dysfunction, *IIEF-5* International Index of Erectile Function (five item)

### Description of population with ED cases

Among all sexually active men, 1,153 (35.1%) responders had IIEF-5-defined ED cases (Fig. 1). Their socio-economic status and comorbidities were described in perspective to the non-ED population (Table 4). The proportion of participants with comorbidities was mostly higher in participants with ED than in those without and increased with age. In young adults to the elderly, hypertension ranged from 5.5 to 41.6% participants with ED vs. 3.5–39.0% without ED; diabetes type I/II ranged from 3.3 to 19.8% participants with ED vs. 1.0–10.2% without ED; high cholesterol ranged from 5.1 to 40.1% participants with ED vs. 4.4–43.2% without ED. The proportion of participants with mental health conditions was mostly higher in participants with ED than in those without, but for both groups, this decreased with age; for example, in young adults to the elderly, mental health conditions ranged from 20.2 to 4.6% in those with ED vs. 11.0–4.2% in those without ED (Supplementary Table S4).

**Table 4:** Socio-demographic and main comorbidities in patients defined with ED (*N* = 1,153) and not defined with ED (*N* = 2,136) according to age group (% weighted)

	Young adult 18 to 34 years (*N* = 955)		Adults 35 to 49 years (*N* = 1,446)		Older adults 50 to64 years (*N* = 573)		Elderly ≥ 65 years (*N* = 315)	
	ED (*N* = 263)	No ED (*N* = 692)	ED (*N* = 438)	No ED (*N* = 1008)	ED (*N* = 255)	No ED (*N* = 318)	ED (*N* = 197)	No ED (*N* = 118)
Race,*n*(%)								
White	129 (58.8%)	478 (76.8%)	281 (65.6%)	742 (75.5%)	186 (72.8%)	252 (79.2%)	163 (82.7%)	104 (88.1%)
Black	43 (19.6%)	74 (11.9%)	66 (16.5%)	105 (11.5%)	28 (12.2%)	36 (12.6%)	18 (9.1%)	7 (5.9%)
Asian	40 (11.0%)	38 (3.7%)	44 (9.8%)	59 (5.7%)	22 (9.6%)	7 (2.5%)	10 (5.1%)	4 (3.4%)
Other/mixed race	43 (10.5%)	89 (7.7%)	44 (8.1%)	92 (9.1%)	16 (5.4%)	21 (5.7%)	6 (3.0%)	3 (2.5%)
**SES category**,** n (%)**								
Low	75 (27.2%)	160 (23.1%)	153 (35.0%)	246 (24.4%)	67 (27.1%)	46 (14.6%)	50 (25.4%)	26 (22.0%)
Middle	344 (48.7%)	114 (44.5%)	172 (39.4%)	453 (45.4%)	127 (48.5%)	181 (56.5%)	99 (50.3%)	50 (42.4%)
High	167 (24.5%)	60 (23.0%)	93 (21.2%)	249 (24.9%)	42 (16.8%)	67 (21.3%)	36 (18.3%)	26 (22.0%)
**Patient comorbidities**,** n (%)**								
At least one comorbidity of interest	102 (38.3%)	150 (22.0%)	215 (49.5%)	379 (37.5%)	186 (73.6%)	186 (58.4%)	159 (80.7%)	83 (70.3%)
Hypertension (high blood pressure)	15 (5.5%)	24 (3.5%)	81 (18.7%)	128 (12.8%)	107 (42.5%)	99 (31.4%)	82 (41.6%)	46 (39.0%)
Diabetes (type I or type II)	9 (3.3%)	7 (1.0%)	38 (8.7%)	42 (4.1%)	49 (19.4%)	23 (6.6%)	39 (19.8%)	12 (10.2%)
High cholesterol	13 (5.1%)	31 (4.4%)	64 (14.6%)	118 (11.6%)	81 (32.4%)	88 (27.6%)	79 (40.1%)	51 (43.2%)
Mental health conditions of any kind (e.g., major depressive disorder, anxiety)	53 (20.2%)	74 (11.0%)	73 (17.0%)	122 (2.1%)	34 (13.6%)	12 (3.8%)	9 (4.6%)	5 (4.2%)

Responses were weighted using the racial/ethnic distribution from the 2021 USA Census by age group

*ED* erectile dysfunction

### Predictors of signs of IIEF-5 defined ED across age groups

**Fig. 2:**
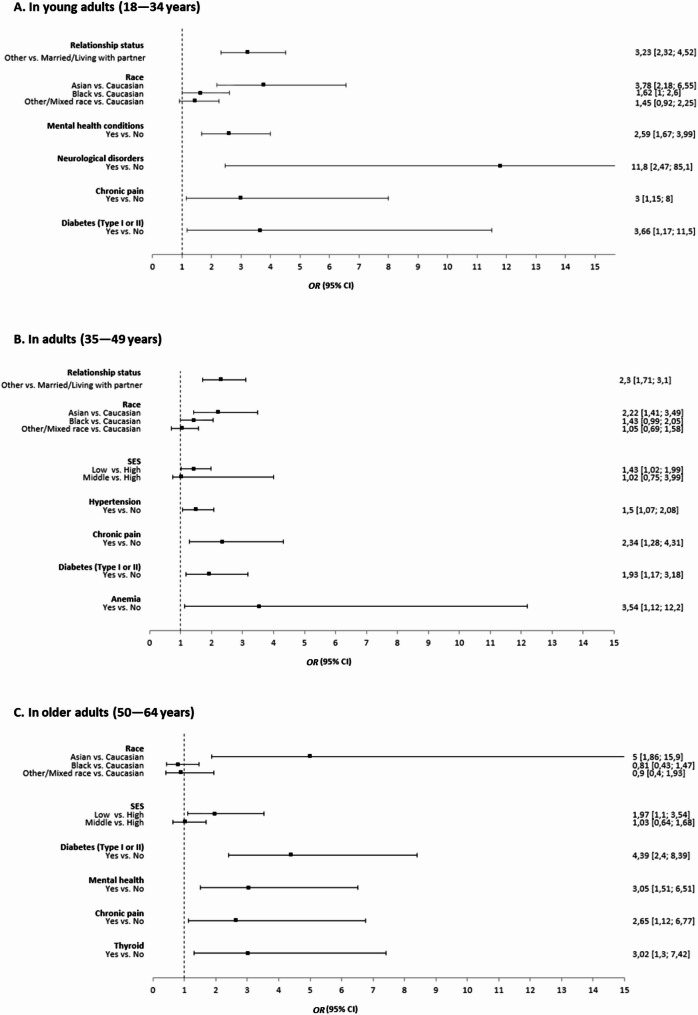
Predictors of IIEF-5-defined ED by age group. CI, confidence interval; ED, erectile dysfunction; OR, odds ratio

The predictors of signs of ED in participants with IIEF-5 defined ED were assessed versus those participants without IIEF-5-defined ED and were reported by age group (Fig. 2). Among young adults (18–34 years old, *N* = 955), the odds of having IIEF-5 defined ED was 3.2 times higher among those who were not married or living with a partner compared to any other relationship status (OR: 3.2 [2.3, 4.5]; *p* < 0.001) (Fig. 2A), 3.8 times higher among Asian participants compared to White participants (OR: 3.8 [2.2, 6.6]; *p* < 0.001) and 1.6 times higher among Black participants compared to White participants (OR: 1.6 [1.0, 2.6]; *p* = 0.05). The odds of having IIEF-5-defined ED were also significantly higher among participants with any mental health condition compared to those without (OR: 2.6 [1.7, 4.0]; *p* < 0.001), those with chronic pain compared to those without (OR: 3.0 [1.2, 8.0]; *p* = 0.03) and those with type I or type II diabetes compared to those without (OR: 3.7 [1.2, 11.5]; *p* = 0.02). Lastly, IIEF-5-defined ED was more than 11.8 times more likely among participants with neurological conditions compared to those without (OR: 11.8 [2.5, 85.1]; *p* = 0.004).

Among adult participants (35–49 years old, *N* = 1,446), the odds of having signs of IIEF-5-defined ED was 2.3 times higher among those who were not married or living with a partner compared to any other relationship status (OR: 2.3 [1.7, 3.1]; *p* < 0.001) (Fig. 2B**)**, and 2.2 times higher among Asian participants compared to White participants (OR: 2.2 [1.4, 3.5]; *p* < 0.001). Low SES was associated with 1.4 higher odd compared to high SES among participants aged 35–49 years (OR: 1.4 [1.0, 2.0]; *p* = 0.04). The presence of hypertension (OR: 1.5 [1.1, 2.1]; *p* = 0.02), chronic pain (OR: 2.3 [1.3, 4.3]; *p* = 0.01) and type I or type II diabetes (OR: 1.9 [1.2, 3.2]; *p* = 0.01) were all significantly associated with higher odds of having IIEF-5-defined ED. Having anaemia of any kind was associated with more than three times the odds of IIEF-5-defined ED compared to not having anaemia (OR: 3.5 [1.1, 12.2]; *p* = 0.03).

Lastly, among older adults (50–64 years old, *N* = 573) (Fig. 2C**)**, Asian participants demonstrated significantly higher odds of IIEF-5-defined ED compared to White participants (OR: 5.0 [1.9, 15.9]; *p* = 0.003). Participants with low SES had two times higher odds of IIEF-5-defined ED compared to participants with high SES (OR: 2.0 [1.1, 3.5]; *p* = 0.02). Significantly higher odds of signs of IIEF-5-defined ED were also associated with having any mental health condition (OR: 3.1 [1.5, 6.5]; *p* = 0.003), chronic pain (OR: 2.7 [1.1, 6.8]; *p* = 0.03) and thyroid disorders (OR: 3.0 [1.3, 7.4]; *p* = 0.01). The odds of having signs of ED was 4.4 times higher among those with type I or type II diabetes compared to those without (OR:4.4 [2.4, 8.4]; *p* < 0.001).

Compared to those without IIEF-5-defined ED, no predictors were significantly associated with a risk of IIEF-5-defined ED signs among participants aged ≥ 65 years.

### Distribution of severity of ED among ED cases

**Fig. 3:**
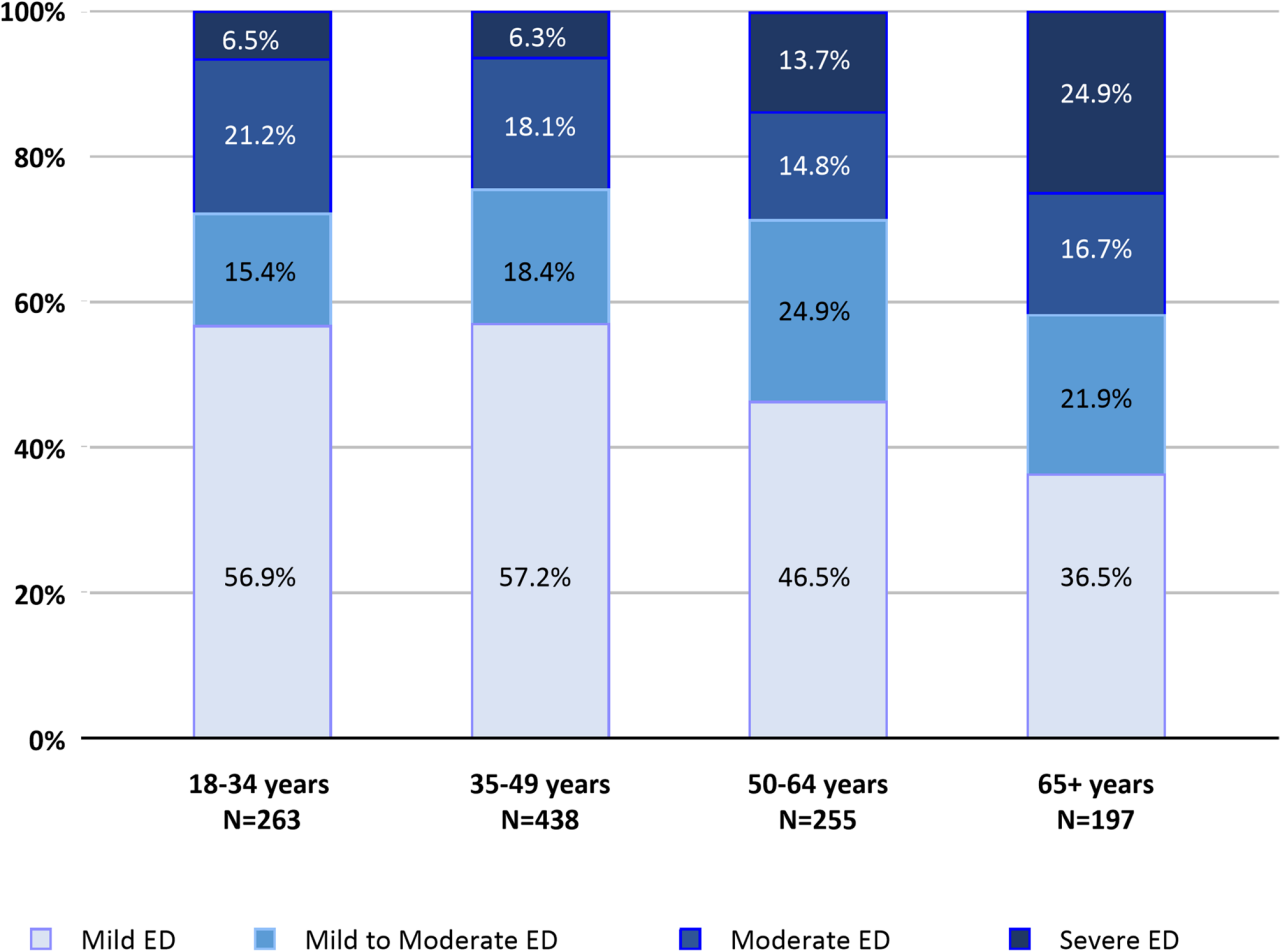
Distribution of ED severity in ED cases among sexually active males according to age group. Total score of 2–7 and sum of questions 15–18 of > 0=severe ED; total score of 8–11=moderate ED; Total score of 12–16=mild to moderate ED; total score of 17–21=mild ED. The numbers of participants in each group are mutually exclusive. ED, erectile dysfunction; IIEF-5, 5-item International Index of Erectile Function

Figure 3 depicts the distribution, by age group, of IIEF-5-defined ED severity (*N* = 1,153). Mild ED was the most common ED severity level among participants regardless of age group and was self -assessed by 57% of young adults and adults. The proportion of participants with severe ED increased with age, ranging from 6.5% among young adults to 24.9% in elderly adults. Moderate and severe ED were experienced by less than one third of young adult participants (27.7%), adults (24.4%) and older adults (28.5%), but it was experienced by 41.6% participants in the oldest age group. The distribution of mild to moderate and moderate ED remained fairly constant across age groups.

### Distribution of self-perception and management among ED cases

**Fig. 4:**
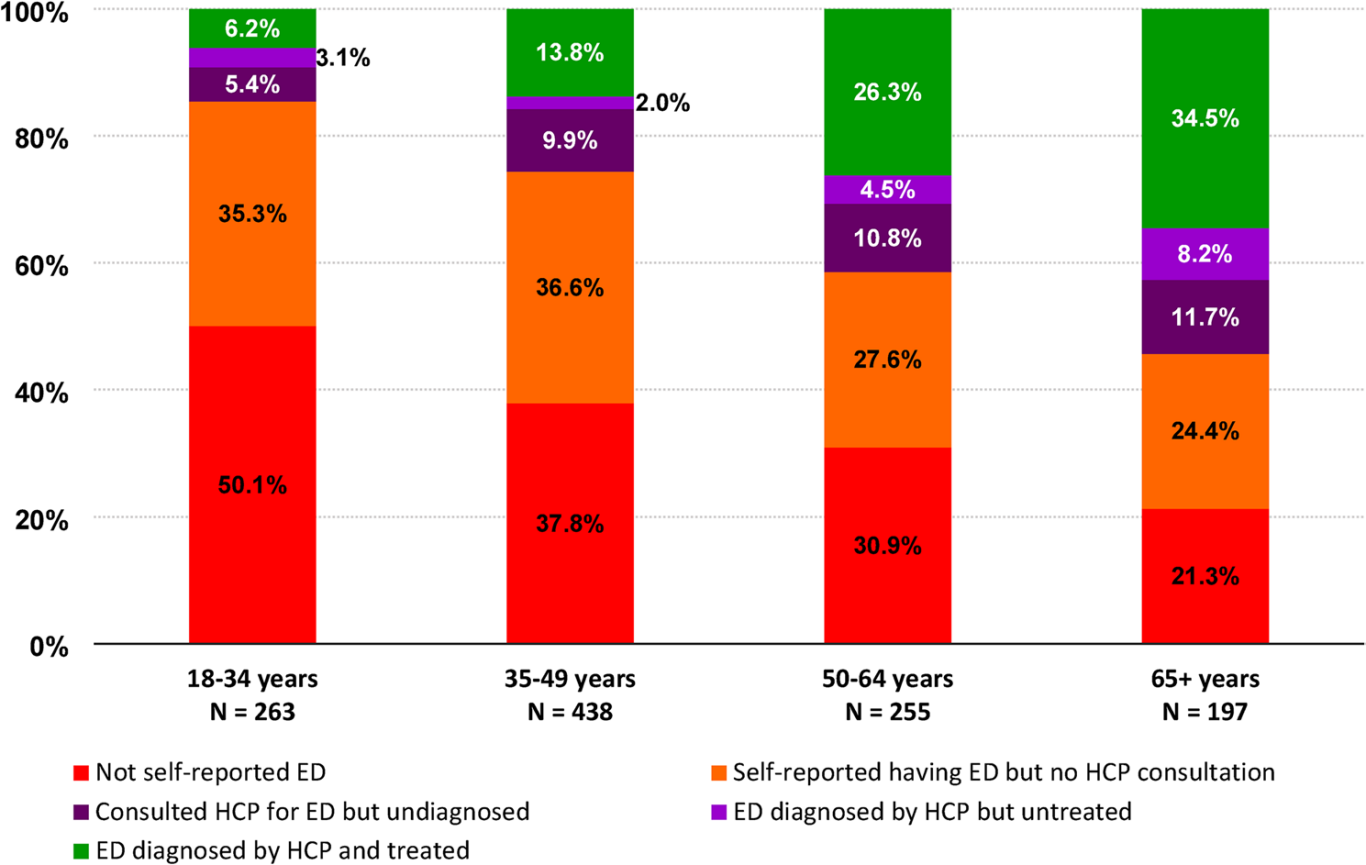
Self-perception and management of ED in ED cases among sexually active males according to age group. The numbers of participants in each group are mutually exclusive. ED, erectile dysfunction; HCP, healthcare professional

Figure 4 depicts the distribution by age group of self-perception and management of ED among all participants with IIEF-5-defined ED (*N* = 1,153). Results showed a lack of awareness of ED, especially in the younger population; about 50% of participants did not self-report signs of ED. Overall, the proportion of self-reported ED decreased across age groups (from 35.4 to 24.3% in the 18–34 years and ≥ 65 years groups, respectively). The lack of awareness was associated with a low medical consultation rate in the young population (35–37% and 24–28% among participants < 50 years and > 50 years respectively). Overall, 85% of young adults and 75% of adults were not aware or did not seek any support for their ED. The proportion of participants who reported any use of ED treatments increased with age, from only ~ 14% of those aged 18–34 years reporting speaking to a healthcare provider (HCP) regarding ED, being diagnosed with ED or treated with ED, compared to ~ 55% of participants aged ≥ 65 years. Overall, ED appropriate management (diagnostic and treatment) constantly increased across age group (from only 6.2–34.5% in the young adult and elderly groups, respectively).

## Discussion

The prevalence of ED varies widely across studies, with global and United States (USA) estimates ranging between 13 and 71% and 18–69%, respectively [4, 5, 7, 11, 15, 32–35]. The wide range is likely reflective of differences in the study population, for example, age, comorbidities, diverse ED definitions and major methodological differences [36]. There is a lack of reliable and precise data on ED prevalence among adult men, highlighted by a systematic review of 41 studies evaluating ED prevalence; 15 studies used the IIEF questionnaire; 10 used MMAS-derived questionnaires, and 16 used other methods [11]. The major strength of our study was the use of one main method, the IIEF-5 questionnaire, alongside two other methods, participant self-perception and ED medication intake, all assessed in the same cohort. Prevalence estimates were assessed across the three methods and alongside existing prevalence estimates in the literature which allowed an understanding of the demographic and clinical profiles of adult males who may under-report or not recognise the early symptoms of ED.

In our study, the overall extrapolated prevalence of ED, based on the IIEF-5 questionnaire, was 39.0%, with an increase observed with advancing age (young adults: 26.0%; adults: 30.3%; older adults: 44.6%; elderly: 62.5%). Age is a well-known risk factor for ED and the majority of men experience ageing-related ED at some point in their lives [10]. Our results confirmed the necessity for an age specific approach in this therapeutic area, as do several other studies [15]: Data from the MMAS showed about 40% of men in their 40 s will have some form of ED increasing by about 10% per decade [8, 15, 37]. Similarly, a cross-national study that enrolled men without comorbid disease showed that advancing age correlated with an increasing overall prevalence of ED [37].

As well, additional factors and predictors associated with an increased risk of ED were identified that differed in some cases across age groups, although in the elderly population (≥ 65 years), no specific factors or comorbidities were found to be associated with the risk of having ED. We found that participants with a low SES had significantly higher odds of ED compared to those with a high SES, this was in agreement with a study using a large, nationally representative USA sample [38]. We also found Asian or Black participants had significantly higher odds of ED compared to participants from other ethnic groups, consistent with results from a similar cross-sectional study showing that Black men demonstrated a higher prevalence of moderate or severe ED (24.4%) compared with White men (21.9%) [5]. We also showed as has already been reported, that the probability of ED increases sharply with comorbidities, and particularly with conditions such as diabetes, cardiovascular disease, hypertension, dyslipidaemia, obesity, metabolic syndrome and depression [9, 18, 39, 40]. We found the type of comorbidities associated with ED differed by age and that hypertension, high cholesterol and diabetes were more prevalent among participants >35 years, whereas mental health conditions and neurological disorders were more prevalent among younger participants.

An important finding in our study, was a potential knowledge deficit about signs and symptoms of ED [41, 42], particularly in younger men (18–34 years) and those with lower SES, requiring specific management strategies facilitating ED awareness and specific access to pharmacological treatment. Within the literature, there is evidence of a gap between patients self-perception of having ED compared with a diagnosis from a validated survey. Takeuchi et al. showed that only 39% of surveyed men aged 40–69 years attending routine visits at a family medicine clinic in Japan reported having sexual dysfunction, but based on the IIEF-5 questionnaire, 92% of them met the ED criteria [41]. Similarly, in a study in Taiwan, 13.1% self-reported ED and 26.0% met the ED criteria using the IIEF-5 questionnaire. In our study, among the ED-diagnosed population, 35% of young adults self-reported having signs of ED, but only 6.2% were diagnosed and treated, whereas 24% of elderly men self-reported having signs of ED and yet 35% diagnosed and treated [42]. Education campaigns and awareness interventions need to target younger men who are most likely to have subtle symptoms of ED and be hesitant to report/manage their ED due to the stigma barrier of this disease.

Our study suggests that mild ED is the most prevalent severity level among participants which is particularly important as mild ED symptoms have the highest risk of being unrecognised, undiagnosed and untreated. Mild ED was most prevalent among young adult participants with ED (57%). Recognising ED early is critical because ED could be a useful marker for comorbid conditions such as cardiovascular disease and diabetes [39]. It is important to note that younger men with several comorbidities have the same risk of ED as healthy men who are 15–25 years older [43]. As well, ED occurs 3–5 years before the signs of cardiovascular disease appear [44]. Indeed, as noted by Rastrelli et al.., the role of ED as a marker of CV risk may be even greater in younger men than in older ones [45].

As well as being underdiagnosed, this study showed that ED often remains undertreated, consistent with other studies [20, 46, 47]. For example, a European study by Jannini et al. found that 68% of men who discussed ED with their healthcare provider were not using treatment [47]. A USA cohort study that looked at ED prevalence and severity in men aged 18–31 years, found that while 14.2% had ED, only 2.0% used either ED treatment or supplements [48].

The availability of drugs, such as sildenafil and tadalafil over the counter, may encourage more men to seek treatment to address their needs and achieve comfort in their sex life. In the 12 months following the reclassification of sildenafil it was associated with a higher number of physician, nurse, or pharmacy visits and higher quality of life. This greater engagement with the healthcare system may facilitate early diagnosis and management of ED and underlying comorbidities [49].

A limitation of our study was that participants with a score ≤ 5 and a sum of questions 15–18 of the study survey equal to 0 were considered as sexually inactive and therefore excluded from the analyses. By doing this, we acknowledge that we were not able to distinguish between those whose sexual functioning was so poor that they did not attempt any sexual activity and those with no desire for nor opportunity to engage in sexual activity. As well, the use of generic alternatives to the branded drugs were not captured in the survey, as in real-world practice, patients typically have insufficient scientific knowledge to accurately identify active pharmaceutical ingredients.

**Fig. 5:**
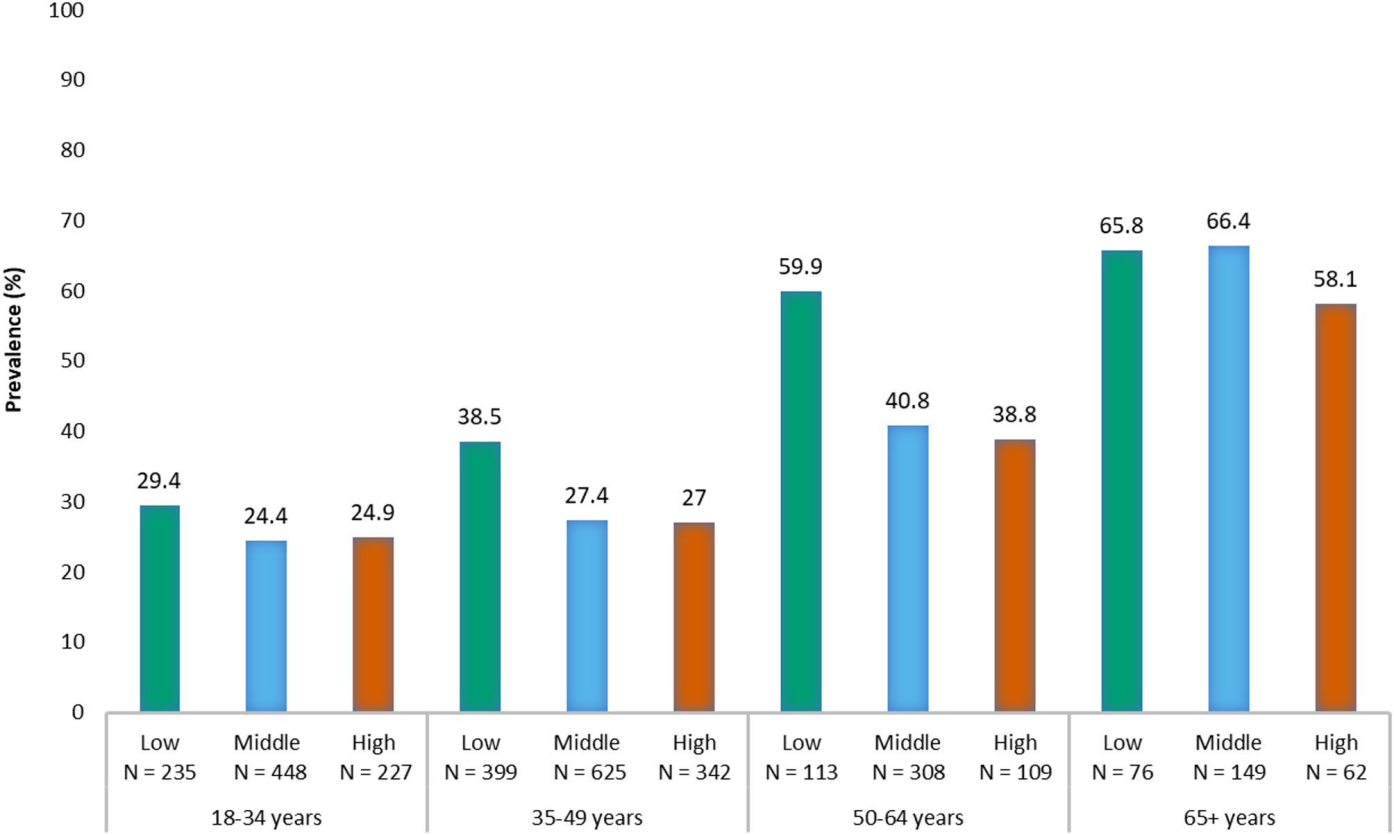
Distribution of ED prevalence (according to age group and socioeconomic status [SES] category. IIEF-5, 5-item International Index of Erectile Function; SES, socioeconomic status

Another limitation of this study is that due to the misunderstanding and poor acceptance of ED due to stigma, the results may be subject to outcome misclassification, thus underestimating the true prevalence of ED in the sample population. In addition, the participants were less diverse racially and ethnically, and held middle to high SES status, compared with the general USA population. To mitigate for the bias in race/ethnicity, panel participants were re-sampled using a proportional weighting strategy based on the racial/ethnic distribution within the 2021 USA Census population. While this may have minimised the effect of selection bias related to race/ethnicity, this did not correct for the fact that the Evidation sample may not have been representative of the USA general population in terms of SES. Because of this, the ranges of SES levels in this study were higher than those defined by the USA 2020 census, which were as follows: lowest quintile: $14,589; second quintile: $39,479; middle quintile: $67,846; fourth quintile: $109,732 and highest quintile: $253,484 [50]. A sensitivity analysis was conducted to assess prevalence estimates by SES index in every age group to evaluate the possible effect of selection bias relating to SES on the sample estimates; it showed higher prevalence among participants with low SES compared to high SES in all age groups, with the largest difference between SES groups found in the older adult group (+ 21.1%). The smallest difference was found in the young adult group (+ 4.5%) (Fig. 5). If, as assumed, the study participants had a higher SES status than the general USA population, the ED prevalence estimates found in this study may have been an underestimation compared to actual ED prevalence in the general population.

## Conclusions

The findings from this study confirm existing knowledge that ED prevalence increases with age, the risk of ED is highly associated with the presence of other comorbidities (although this finding is without statistical significance among participants aged ≥ 65 years) and is influenced by socio-economic factors. Additionally, this study provides the evidence of slight discordance between participants with IIEF-5-defined ED and those who self-reported their signs of ED. This discord was more frequent in younger men having mild symptoms, suggesting a higher risk of being unrecognised, undiagnosed and untreated for their ED. This group is least likely to seek medical care for ED symptoms and are also less likely to receive systematic screening even though they may benefit the most from the recognition that ED is an early indicator for more serious health conditions.

## Supplementary Information

Supplementary Material 1.

## Data Availability

Qualified researchers may request access to patient level data and related study documents including the clinical study report, study protocol with any amendments, blank case report form, statistical analysis plan, and dataset specifications. Patient level data will be anonymized, and study documents will be redacted to protect the privacy of our trial participants. Further details on Sanofi’s data sharing criteria, eligible studies, and process for requesting access can be found at: https://vivli.org/.
