# Supplementary material for: A population-based survey of self-reported and IIEF-defined erectile dysfunction among adult men in the United States in 2021
Source: BMC Public Health. 2025 Nov 27;25:4172. doi: 10.1186/s12889-025-24808-4 (PMC12659121; doi:10.1186/s12889-025-24808-4)
Supplement: Supplementary file 1 — Supplementary Material 1. [file 12889_2025_24808_MOESM1_ESM.docx]

Supplementary Information

Supplementary Table 1. Comorbidities of the sexually active men population by age group (% weighted)

|  | **Young adult  18 to 34 years (N=955)** | **Adults  35 to 49 years (N=1446)** | **Older adults 50 to 64 years (N=573)** | **Elderly ≥65 years (N=315)** | **Overall (N=3289)** |
| --- | --- | --- | --- | --- | --- |
| **At least one comorbidity of interest, n(%)** | 252 (26.4%) | 594 (41.1%) | 372 (64.9%) | 242 (76.8%) | 1,460 (44.4%) |
| **Top 5 comorbidities (ever diagnosed by a healthcare provider), n(%)** |  |  |  |  |  |
| Hypertension (high blood pressure) | 39 (4.1%) | 209 (14.5%) | 206 (36.0%) | 128 (40.6%) | 582 (17.7%) |
| High cholesterol | 44 (4.6%) | 182 (12.6%) | 169 (29.5%) | 130 (41.3%) | 525 (16.0%) |
| Mental health conditions of any kind (e.g., major depressive disorder, anxiety) | 127 (13.3%) | 195 (13.5%) | 46 (8.0%) | 14 (4.4%) | 382 (11.6%) |
| Diabetes (type I/II) | 16 (1.7%) | 80 (5.5%) | 72 (12.6%) | 51 (16.2%) | 219 (6.7%) |
| Chronic pain | 23 (2.4%) | 50 (3.5%) | 34 (5.9%) | 16 (5.1%) | 123 (3.7%) |
| **Other comorbidities (ever diagnosed by a healthcare provider), n(%)** |  |  |  |  |  |
| Heart disease | 12 (1.3%) | 19 (1.3%) | 18 (3.1%) | 31 (9.8%) | 80 (2.4%) |
| Benign prostatic hyperplasia | 1 (0.1%) | 3 (0.2%) | 9 (1.6%) | 25 (7.9%) | 38 (1.2%) |
| Arthritis | 8 (0.8%) | 20 (1.4%) | 35 (6.1%) | 40 (12.7%) | 103 (3.1%) |
| Osteoarthritis | 6 (0.6%) | 15 (1.0%) | 26 (4.5%) | 30 (9.5%) | 77 (2.3%) |
| Rheumatoid arthritis | 3 (0.3%) | 7 (0.5%) | 10 (1.7%) | 11 (3.5%) | 31 (0.9%) |
| Thyroid disorders of any kind (e.g., hyper- or hypothyroidism) | 15 (1.6%) | 43 (3.0%) | 30 (5.2%) | 22 (7.0%) | 110 (3.3%) |
| Cancer of any kind | 9 (0.9%) | 25 (1.7%) | 25 (4.4%) | 30 (9.5%) | 89 (2.7%) |
| Atopic dermatitis | 18 (1.9%) | 23 (1.6%) | 7 (1.2%) | 5 (1.6%) | 53 (1.6%) |
| Neurological disorders of any kind (e.g., Alzheimer’s disease, Parkinson’s disease, stroke, epilepsy or seizures) | 10 (1.0%) | 20 (1.4%) | 10 (1.7%) | 4 (1.3%) | 44 (1.3%) |
| Atrial fibrillation | 4 (0.4%) | 12 (0.8%) | 16 (2.8%) | 22 (7.0%) | 54 (1.6%) |
| Kidney disease of any kind (e.g., chronic kidney disease, renal insufficiency) | 4 (0.4%) | 14 (1.0%) | 9 (1.6%) | 12 (3.8%) | 39 (1.2%) |
| Anaemia of any kind (e.g., thalassaemia or iron-deficiency anaemia) | 6 (0.6%) | 15 (1.0%) | 3 (0.5%) | 8 (2.5%) | 32 (1.0%) |
| Glaucoma | 3 (0.4%) | 8 (0.6%) | 6 (1.1%) | 15 (4.8%) | 32 (1.0%) |
| Ulcerative colitis | 5 (0.6%) | 13 (0.9%) | 2 (0.4%) | 3 (1.0%) | 23 (0.7%) |
| Crohn’s disease | 8 (0.8%) | 7 (0.5%) | 2 (0.3%) | 2 (0.6%) | 19 (0.6%) |
| Celiac disease | 7 (0.7%) | 6 (0.4%) | 1 (0.2%) | 0 (0.0%) | 14 (0.4%) |
| Osteoporosis | 1 (0.1%) | 0 (0.0%) | 5 (0.9%) | 3 (1.0%) | 9 (0.3%) |
| Cystic fibrosis | 2 (0.2%) | 1 0(.1%) | 0 (0.0%) | 1 (0.3%) | 4 (0.1%) |

*Responses were weighted using the racial/ethnic distribution from the 2021 USA Census by age group*
